# The MYH9 Cytoskeletal Protein Is a Novel Corepressor of Androgen Receptors

**DOI:** 10.3389/fonc.2021.641496

**Published:** 2021-04-01

**Authors:** Chunhua Liu, Zhaoping Liao, Xiuzhi Duan, Pan Yu, Piaoping Kong, Zhihua Tao, Weiwei Liu

**Affiliations:** ^1^ Department of Blood Transfusion, The Second Affiliated Hospital of Zhejiang University School of Medicine, Hangzhou, China; ^2^ Department of Laboratory Medicine, The Second Affiliated Hospital of Zhejiang University School of Medicine, Hangzhou, China

**Keywords:** prostate cancer, androgen receptor, MYH9, coregulators, nuclear translocation, transcription regulation

## Abstract

In the progression of castration-resistant prostate cancer (CRPC), the androgen receptor (AR) that serves as a transcription factor becomes the most remarkable molecule. The transcriptional activity of AR is regulated by various coregulators. As a result, altered expression levels, an aberrant location or activities of coregulators promote the development of prostate cancer. We describe herein results showing that compared with androgen-dependent prostate cancer (ADPC) cells, AR nuclear translocation capability is enhanced in androgen-independent prostate cancer (AIPC) cells. To gain insight into whether AR coregulators are responsible for AR translocation capability, we performed coimmunoprecipitation (CO-IP) coupled with LC-MS/MS to screen 27 previously reported AR cofactors and 46 candidate AR cofactors. Furthermore, one candidate, myosin heavy chain 9 (MYH9), was identified and verified as a novel AR cofactor. Interestingly, the distribution of MYH9 was in both the cytoplasmic and nuclear compartments yet was enriched in the nucleus when AR was knocked down by AR shRNA, suggesting that the nuclear translocation of MYH9 was negatively regulated by AR. In addition, we found that blebbistatin, an inhibitor of MYH9, not only promoted AR nuclear translocation but also enhanced the expression of the AR target gene PSA, which indicates that MYH9 represses nuclear AR signaling. Taken together, our findings reveal that MYH9 appears to be a novel corepressor of AR plays a pivotal role in the progression of CRPC.

## Introduction

Despite decreasing rates of some cancers amenable to early detection, prostate cancer (PCa) is the second deadliest cancer and the incidence remains stubbornly the highest in the American for over two decades (1). Androgen deprivation therapy (ADT) is the most frequent therapy for alleviating prostate cancer progression (2). Nonetheless, once cancer cells ultimately survive from androgen deprivation, they evolve to a highly aggressive androgen-independent phase, which is termed androgen-independent prostate cancer (AIPC) or castration-resistant prostate cancer (CRPC) (3). Generally, angiogenesis in CRPC is strongly enhanced, which promotes migration and metastases of tumor cells (4). The mechanisms underlying the evolving of prostate cancer to androgen independence remain largely unclear. Therefore, unraveling the mechanisms of androgen insensitivity in PCa may provide new targets for effective treatment of CRPC.

In the progression of CRPC, AR that serves as a transcription factor, becomes the most remarkable molecule. Interestingly, AIPC cells continuously rely on AR and its signaling cascades, whereas other pathways such as alternative pathways also promote PCa to a more aggressive stage (5). The aberrant regulation of AR, such as mutations, gene amplification, and enzymes that catalyze the synthesis of ligand or disturbed coregulators, results in the development of AIPC (6, 7). On the other hand, AR transcriptional activity can also be activated by a number of different proteins through outlaw pathways which is independent of ligand (8). Insulin-like growth factor-1 (IGF1) is one of the extremely investigated proteins, which stimulates AR nuclear translocation, binding to the promoter of target genes and initiating transactivation in undetectable androgen conditions (9). Regardless of AR signaling, bypass pathways that independent of AR have also been involved in the progression of AIPC; for example, GR bypasses AR and acts directly on AR target gene glucocorticoid-induced protein kinase 1 (SGK1) expression, which is sufficient to keep androgen-independent proliferation of PCa cells (10, 11). When AR binds to an androgen, it formulates dimerization, imports to the nucleus, anchored to the promotor of target genes and initiates transcription; however, it form a complex with chaperone proteins and is retained in the cytoplasm without androgen (5).

AR requires functional and structural interactions with other transcriptional regulators (coactivators or corepressors), which can either enhance or reduce AR transactivation. AR coactivators, mainly including the p160 steroid receptor coactivator (SRC) family (SRC1, SRC2 and SRC3), chaperone proteins, filamentous actin (f-actin)-binding proteins, DNA repair proteins and ubiquitin ligases, were crucial for PCa progression as well as AIPC conversion (12, 13). One of AR coactivators, semenogelin I, interacts with AR when treated with Zn^2+^ leading to a significant enhancement of growth in PCa cells (14). In addition, the two well-known corepressors, nuclear receptor corepressor (NCoR) and silencing mediator of retinoid and thyroid hormone receptor (SMRT), complexed with heat shock proteins, preventing AR binding to target genes, therefore resulting in repressive effects (14). Although AR coregulators have been comprehensively investigated in PCa (15, 16), their role and mechanism in AIPC remain unclear.

To mimic patients undergoing ADT treatment, we had already generated two androgen-independent cell lines, LNCaP-AI cells and LNCaP-AI+F cells derived from androgen-sensitive LNCaP cells cultured under androgen-depleted conditions (17). Our previous study demonstrated that the proliferation and invasion abilities of LNCaP-AI cells were significantly enhanced compared to LNCaP cells (18). In this study, we discovered that the AR nuclear translocation capability was enhanced in LNCaP-AI cells compared to LNCaP cells, which resulted in LNCaP-AI cells proliferation and invasiveness, further confirming our previous observations (18). Furthermore, different AR cofactors between ADPC and AIPC cells were investigated using CO-IP coupled with LC-MS/MS. Interestingly, myosin heavy chain 9 (MYH9) was identified and demonstrated to be a novel corepressor of AR.

## Materials and Methods

### Cell Culture and Reagents

LNCaP cells, a typical androgen-dependent prostate cancer cell line, were purchased from the Cell Bank of Shanghai Institute of Biochemistry and Cell Biology, Chinese Academy of Sciences (Shanghai, China); LNCaP-AI cells, an androgen-independent cell line, were generated as previously described (17). LNCaP cells were cultured routinely in Ham’s F12 (F12) medium with 10% fetal bovine serum (FBS; Gibco Life Technologies, Carlsbad, CA, USA) at 37°C in 5% CO_2_, while LNCaP−AI cells were grown in phenol red−free Dulbecco’s modified Eagle’s medium/Ham’s F12 (DMEM/F12) medium with 10% dextran−charcoal stripped FBS (CS−FBS; Gibco Life Technologies). Another cell line, LNCaP-AI+F, was induced as LNCaP-AI cells but was maintained in phenol red−free DMEM/F12 with 10% CS−FBS containing 10 μM hydroxyflutamide for more than 100 passages and maintain throughout all experiments. DHT (A8380, Sigma), hydroxyflutamide tablets (Forward, Shanghai, China) and (-)-blebbistatin (S7079, Selleck) were commercially obtained. DHT was dissolved in alcohol at a concentration of 10 mM as a stock solution and was kept in a light-resistant container. Hydroxyflutamide tablets were also dissolved in alcohol at a concentration of 10 μM. Blebbistatin was dissolved in DMSO at a final concentration of 10 μM.

### RT-qPCR

Total RNA was extracted from 10 nM dihydrotestosterone (DHT)-treated or equivalent ethanol-treated cells for the indicated times using TRIzol Reagent (15596-018, Invitrogen Corporation, USA) according to the manufacturer’s instructions. For reverse transcription, 1 μg of total RNA was used with a Transcriptor First Strand cDNA Synthesis Kit (04379012001, Roche Applied Science, USA) according to the manufacturer’s procedure. mRNA expression was analyzed by quantitative reverse transcription-PCR (RT-qPCR) using FS Universal SYBR Green Master Mix (4913850001, Roche Applied Science, USA). The following primers were applied in this study:

AR Forward: 5-’GCCACTCAGACCCACTTAGC-3’
AR Reverse: 5-’CCTCACTCTTCGTCCACATCG-3’
PSA Forward: 5-’CCTAGATGAAGTCTCCATGAGCTAC-3’
PSA Reverse: 5-’GGGAGGGAGAGCTAGCACTTG-3’
MYH9 Forward: 5-’ACAGGTCGTCGGACCGAGAA-3’
MYH9 Reverse: 5-’TAGGAGCTGACTTCGCGGT-3’
GAPDH Forward: 5-’CTGAGCACCAGGTGGTCTC-3’
GAPDH Reverse: 5-’AGGGACTCCCCAGCAGTGAG-3’

ABI 7500 Real-Time PCR system (Applied Biosystems, Foster City, CA, USA) was used to perform qPCR. Three independent samples were collected in triplicate and analyzed using 2^-ΔΔCt^ relative quantitative analysis.

### Nuclear and Cytoplasmic Extraction

Collected cells were rinsed twice with precooled PBS and extracted by NE-PER™ Nuclear and Cytoplasmic Extraction Reagents (78835, Thermo Scientific) strictly in accordance with the instructions. After lysing in CERII combined with proteinase inhibitor cocktail (B14011, Selleck), the samples were centrifuged at 16,000 rpm for 10 min, and the supernatant was collected as the cytoplasmic fraction. Likewise, the nuclear extract was collected after NER was added.

### Coimmunoprecipitation Assay

Three PCa cell lines were seeded for 24 h in FBS (LNCaP cells) or CS-FBS (LNCaP-AI cells) or CS-FBS added with 10 μM hydroxyflutamide (LNCaP-AI+F cells) before 10 nM DHT or equal amount of ethanol treatment and harvested at the indicated time points (72 h for LNCaP cells, 48 h for LNCaP-AI and LNCaP-AI+F cells). Five hundred micrograms of total protein extracted by RIPA lysis buffer (Beyolife, China) were immunoprecipitated with AR or MYH9 antibodies. Control immunoprecipitation was performed using rabbit normal IgG (Millipore Corporation). The antigen-antibody mixtures protected by proteinase inhibitor cocktail were incubated with gentle rotation overnight at 4°C. The antigen-antibody complexes were incubated with PureProteome Protein A Magnetic Beads (LSKMAGA02, Millipore) (2 μl of beads to 1 μg of antibody) at 4°C for 2 hours with gentle rotation and then separated by a magnetic frame. After washing three times with 500 µl of 1×PBS (pH 7.4) containing 0.1% Tween-20, the pull-down complexes were eluted by 60 μl of citrate buffer (1 M, pH 10.0) and neutralized by 5 µl of glycine buffer (1 M, pH 2.4) for LC/MS/MS detection or were eluted by boiling the beads in 5× loading buffer (50 mM Tris, pH 6.8; 2% SDS; 10% glycerol; 2.5% beta-mercaptoethanol; and 0.02% bromophenol blue) and were subjected to western blotting or Coomassie brilliant blue staining (R250, Beyotime Biotechnology, China).

### Western Blot Analysis

Proteins were prepared from (1) whole-cell lysates of LNCaP or LNCaP-AI cells with or without 10 nM DHT treatment; (2) cytosolic or nuclear fraction extracts; and (3) CO-IP elution. Proteins were diluted with 5× loading buffer. Thirty microliters of each diluted sample were boiled for five minutes, separated on 10% SDS-PAGE gels, and transferred to PVDF membranes (0.22 μM, ISEQ00010, Millipore Corporation). The membranes were blocked with 5% bull serum albumin for two hours at room temperature, incubated with primary antibodies recognizing AR (rabbit, ab74272, Abcam), MYH9 (mouse, ab55456, Abcam), or GAPDH (rabbit, 2118, CST) overnight at 4°C, and finally detected with anti-rabbit IgG-HRP (7074, Cell Signaling Technology, USA) or anti-mouse IgG-HRP (7076, Cell Signaling Technology, USA). The protein bands were visualized using chemiluminescent HRP substrate (Millipore), followed by exposure to ChemiDoc (Bio-Rad).

### CO-IP Complex Identification

The eluate mentioned above was electrophoresed by SDS-PAGE, digested in gel and eluted before being loaded for liquid chromatography coupled with tandem mass spectrometry (LC−MS/MS) (Triple TOF 5600, Shimadzu) detection. MS/MS spectra were processed and queried against the NCBInr protein database with taxonomy filter set for Homo sapiens (91464 sequences) using the MASCOT algorithm (https://www.matrixscience.com) for protein identification. Search parameters for MS/MS database were as follows: one missed cleavage in the trypsin digests was allowed and a mass tolerance of 0.05 Da (the tolerance was “± 0.05 Da”) was permitted for intact peptide masses and ±0.1 Da for fragmented ions. Potential variable modifications were Gln → pyro-Glu (N-term Q), Oxidation (M), Deamidated (NQ) and Carbamidomethyl (C) as fixed modifications. Charge distribution of the peptides were set to +2, +3 and +4. Qualitative data analysis was performed with MASCOT using a 99% confidence interval. We report protein scores as Mascot score (-10*LOG10(P), where P is the absolute probability). For example, a 1% probability that the peptide spectrum match is a random event would translate into a Mascot score of 20. Cluster of orthologous groups of proteins (COG, https://www.ncbi.nlm.nih.gov/research/cog), a system for automated detection of homologues among the annotated genes of several completely sequenced eukaryotic genomes, were applied for functionally annotation and classification of all the identified proteins. We mapped the cofactor-interacting proteins to the protein-protein interaction (PPI) network from the String (https://string-db.org/) website and extracted all cofactor-protein interactions. All proteins in the MS results were screened by text mining for known AR cofactors and possible AR cofactors.

### Immunofluorescence Staining

Cells on coverslips (Eppendorf Scientific, Germany) were washed briefly in PBS and fixed for 10 min in cold 4% paraformaldehyde. Each step was followed by three washes with PBS throughout the procedure. Cells were treated with 0.1% Triton X-100 (Biosharp) for 10 min at room temperature. Blocking was performed for 60 min at room temperature in 10% normal goat serum in PBS, followed by incubation overnight at 4°C with AR or MYH9 antibody at 1:200 in 1.5% goat serum in PBS. The secondary antibody was DyLight 488 goat anti-rabbit IgG conjugate (A23220, Abbkine) or DyLight 594 anti-mouse IgG conjugate (A23410, Abbkine) at 1:1,000 in 1.5% goat serum in PBS and incubated in the dark for 60 min at room temperature. Coverslips were then rinsed three times with PBS and incubated in DAPI (2 mg/L stock solution, D21490, Invitrogen) at 1:1,000 in PBS for 5 min in the dark. The coverslips were carefully removed, rinsed briefly with ddH_2_O to remove unbound dye and mounted in ProLong (Molecular Probes, Inc.). Finally, immunofluorescence was observed with a fluorescence microscope (BX-51, Olympus, Japan).

### Transfection With shRNA Lentiviral Particles

AR RNA interference assays were performed as previously described (18). Briefly, ARshRNA lentiviral vectors were obtained from GenePharma Biotechnology, Inc. (Shanghai, China). The forward AR shRNA and scrambled sequence were as followed: 5’-GGAACTCGATCGTATCATTGC-3’ and 5’-TTCTCCGAACGTGTCACGT-3’ respectively. Green fluorescence protein (GFP) expression determined by a FACSCalibur (BD Biosciences) flow cytometer was used for assessment of the transfection efficiency. The efficiency of shRNA interference was evaluated by RT-qPCR and WB analyses.

### Statistics

The values were expressed as the means ± S.D. for triplicate experiments. Date analysis was performed using one-way ANOVA followed by Dunnett’s test for more than three groups or LSD (least significance difference) test for three groups and Student’s two-tailed t-test. Statistical analysis was performed by using SPSS 22.0 (SPSS Inc., Chicago, IL, USA) and a value of P<0.05 or 0.01 was accepted statistically significant.

### Data Processing and Analysis

Colocalization and quantification of fluorescence together with density of protein bands were analyzed with ImageJ 1.52i software (National Institutes of Health, USA). Spots of poor quality in fluorescence pictures were removed from further analysis by visual inspection. Pearson’s R value (above threshold) was applied as colocalization level for AR and MYH9.

## Results

### Discordant Expression of AR and PSA in the Three PCa Cells

**Figure 1:**
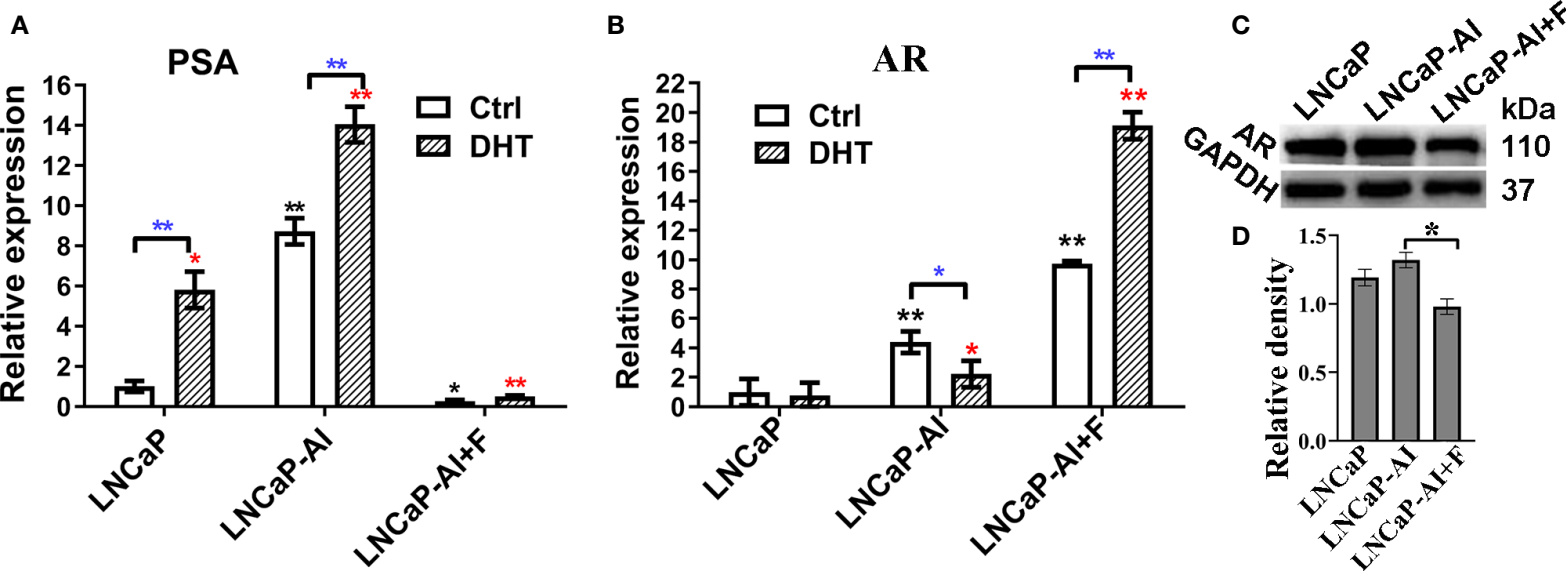
Expression of AR and PSA in PCa cells. **(A, B)** Relative PSA and AR mRNA expression is shown in PCa cell lines tested by qRT-PCR. LNCaP cells were cultured for 3 days while LNCaP-AI cells cultured for 2 days in CS-FBS medium treated with ethanol or 10 nM DHT. The results are expressed as the mean ± S.D. of three biological replicates and normalized to GAPDH. *, P<0.05 or **P<0.01 vs LNCaP cells treated with alcohol or 10 nM DHT; one-way ANOVA followed by LSD multiple comparison test among three cell lines or T test for the same cell line treated with alcohol or 10 nM DHT. **(C, D)** AR protein expression in the three PCa cells. **(C)** Total AR expression in the three PCa cell lines is shown using WB. **(D)** The density of each AR band in three independent experiments of **(C)** was determined (*P < 0.05; one-way ANOVA followed by LSD multiple comparison test, n=3).

Our previous study demonstrated that LNCaP-AI cells undergone long-term androgen deprivation exhibited higher proliferation rate and more aggressiveness than LNCaP cells (18). Additionally, consistent with our previous results, the PSA mRNA was up-regulated in LNCaP-AI cells compared to LNCaP cells regardless of DHT treatment (**Figure 1A**). Interestingly, AR mRNA was significantly higher in LNCaP-AI cells than in LNCaP cells but highest in LNCaP-AI+F cells (**Figure 1B**). AR mRNA was downregulated followed by 10 nM DHT treatment in LNCaP-AI cells while upregulated in LNCaP-AI +F cells. Though AR mRNA was significantly higher in LNCaP-AI +F cells than the other two cells, the PSA mRNA expression was reduced, which was an intriguing phenomenon. What’s more, AR protein expression was similar in LNCaP and LNCaP-AI cells while significantly reduced in LNCaP-AI +F cells (**Figures 1C, D**), and there was no significant increase in AR protein expression after treatment with androgen in LNCaP and LNCaP-AI cells (18).

### AR Nuclear Translocation Capability Was Enhanced in AIPC Cells

**Figure 2:**
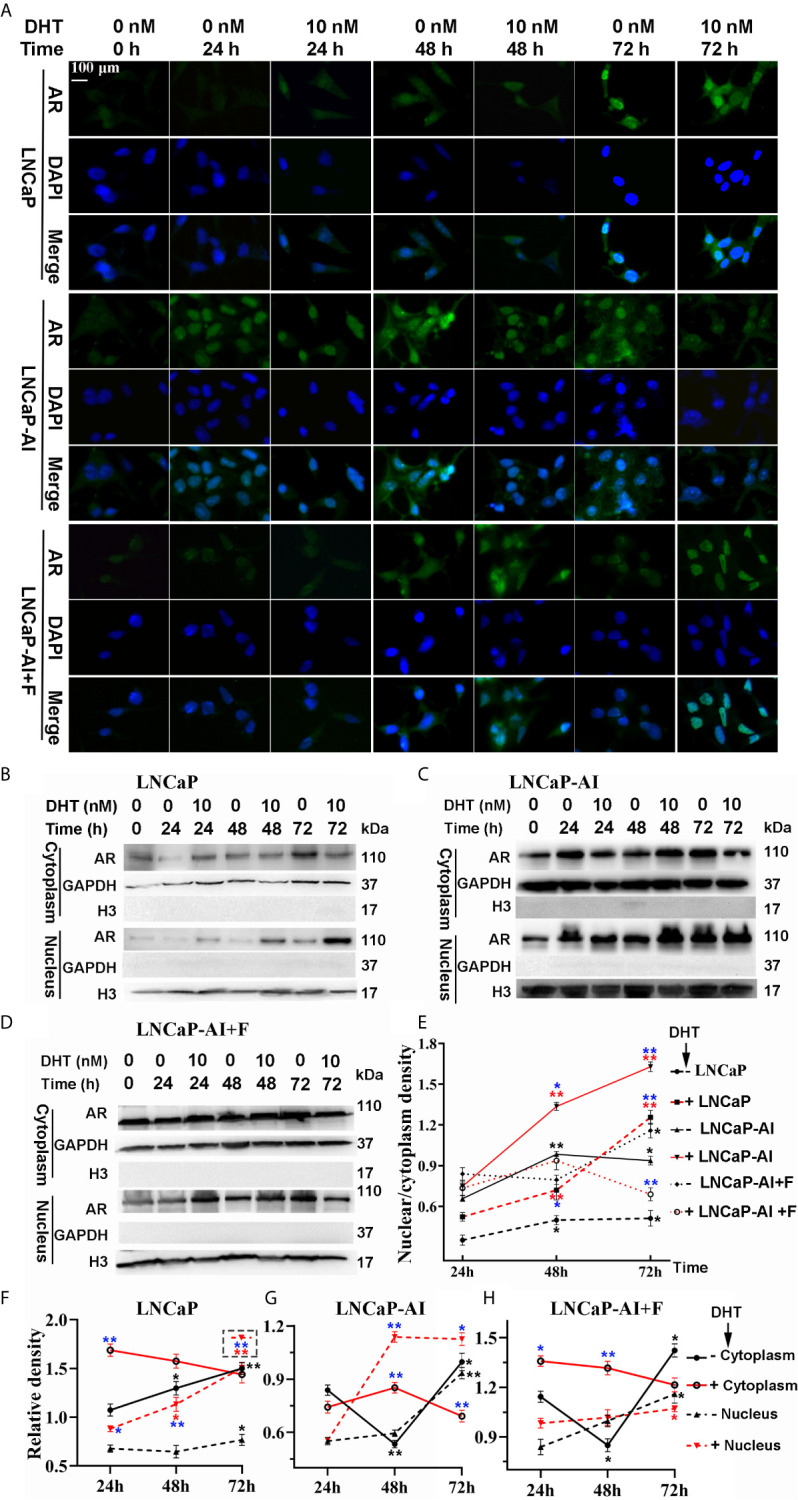
LNCaP-AI cells exhibit higher nuclear translocation capability. **(A–E)** Three PCa cell lines were seeded for 24 h in FBS (LNCaP cells) or CS-FBS (LNCaP-AI cells) or CS-FBS added with 10 μM hydroxyflutamide (LNCaP-AI+F cells), then treated with 10 nM DHT or equal amounts of ethanol and harvested 24 h, 48 h or 72 h latter. AR expression is observed by immunofluorescence **(A)** or WB **(B–D)**. **(A)** Cells were stained for AR (green), DAPI (blue). The scale bar in the upper left corner is 100 μm (40×). **(B–D)** AR cytoplasmic and nuclear extracts are shown in LNCaP, LNCaP-AI and LNCaP-AI+F cells, respectively. **(E–H)** The AR density of each band in **(B–D)** was analyzed by ImageJ 1.52i software (National Institutes of Health, USA). AR levels in each cell line were normalized to GAPDH (cytoplasm) or H3 (nucleus) and shown in **(F–H)** as well as AR nucleus/cytoplasm ratios shown in **(E)**. The results are expressed as the mean ± S.D. of three biological replicates.One-way ANOVA followed by Dunnett’s test for different time points compared with 24 h treated with DHT (*P < 0.05, **P < 0.01) or alcohol (*P < 0.05, **P < 0.01) (n = 2) and T test for the same cell line treated with alcohol or 10 nM DHT (*P < 0.05 , **P < 0.01).

Upregulated expression of PSA indicates that AR signaling pathways are activated, suggesting that not AR expression but AR nuclear translocation is potentially enhanced in LNCaP-AI cells. We consequently analyzed whether AR translocation activity was enhanced in LNCaP-AI cells compared to LNCaP cells. The cells were subcultured in media supplemented with CS−FBS for 24 h (0 h) then treated with DHT or ethanol as a vehicle control for the indicated time (24 h, 48 h, 72 h) before AR was observed by both immunofluorescence microscopy (**Figure 2A**) and western blotting (**Figures 2B–D**). As expected, DHT promoted AR nuclear localization in LNCaP and LNCaP-AI cells which was consistent with PSA expression. However, whether DHT promotes AR nuclear translocation in LNCaP-AI+F cells was intricate. Moreover, nuclear AR accumulation in AIPC cells (especially in LNCaP-AI cells) occurred approximately 24 h earlier than that in LNCaP cells. Similarly, the amount of nuclear AR in LNCaP-AI cells was much higher than that in LNCaP cells at the same time point after DHT or ethanol treatment. Besides, the nucleus/cytoplasm ratios of AR in LNCaP cells treated with 10 nM DHT catch up with and surpass LNCaP-AI+F cells in 72 h later, suggesting LNCaP-AI+F cells was not so sensitivity to DHT stimulation as LNCaP cells. Collectively, our results indicated that the AR nuclear translocation capability was enhanced in LNCaP-AI cells compared to LNCaP cells, the mechanism of which needs further investigation.

### Identification and Screening for AR Cofactors in the Three PCa Cells

**Figure 3:**
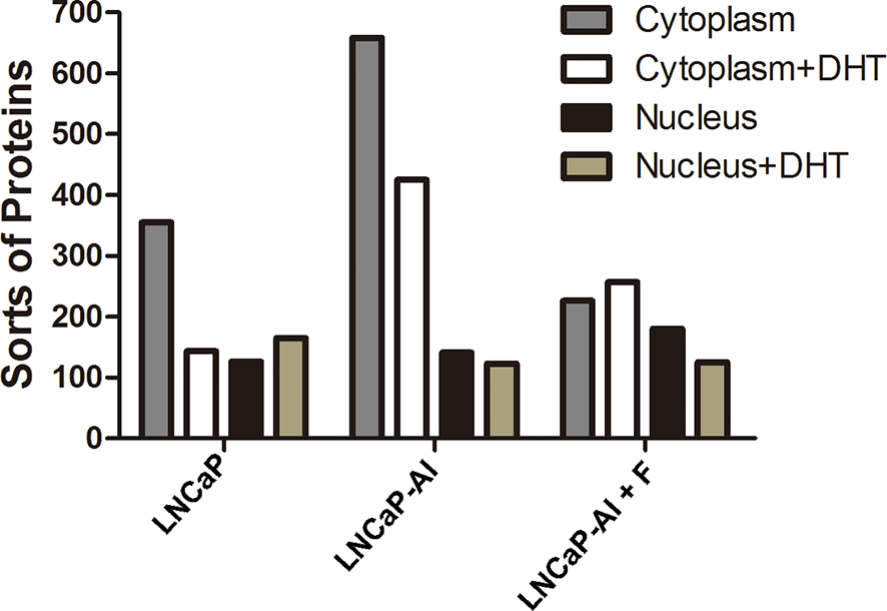
Number of proteins in AR pull-down produces among the three PCa cell lines. Three PCa cell lines were seeded for 24 h before treated with or without 10 nM DHT and harvested at the indicated time points (72 h for LNCaP cells, 48 h for LNCaP-AI and LNCaP-AI+F cells). Cell lysates of cytoplasmic and nuclear proteins were immunoprecipitated using AR McAb and identified by LC-MS/MS.

A variety of studies have confirmed that AR coregulators are closely related to AR nuclear translocation (19); however, the relationship between AR coregulators and AIPC development is still unclear. Therefore, to test whether AR coregulators are responsible for enhanced AR nuclear translocation, CO-IP and LC/MS/MS were performed to search for different AR cofactors between ADPC and AIPC cells. According to the AR translocation phenomenon shown in **Figure 2**, cells were seeded for 24 h, then collected at the indicated time points after 10 nM DHT or the equivalent amount of ethanol was added (72 h for LNCaP cells, 48 h for LNCaP-AI and LNCaP-AI+F cells). Cytoplasmic and nuclear protein lysates of LNCaP cells, LNCaP-AI cells and LNCaP-AI+F cells were immunoprecipitated by AR monoclonal antibody followed by in gel digestion coupled with LC-MS/MS identification. Number of proteins identified in the 12 samples are listed in **Figure 3**. Our results showed that the number of proteins in each sample identified by MS varied from 123 to 658, and there were more sorts of proteins identified in the cytoplasm than in the nucleus. However, it was notable that a markedly greater number of proteins were identified in the cytoplasm of LNCaP-AI cells than in the other two cell lines, which indicates more proteins involved in cytoplasmic AR modulation in LNCaP-AI cells. Generally, the greater the number of proteins identified, the greater the number signaling pathways in which they may be involved. Our results strongly indicate that AR signaling pathways are more intricately modulated in LNCaP-AI cells.

**Figure 4:**
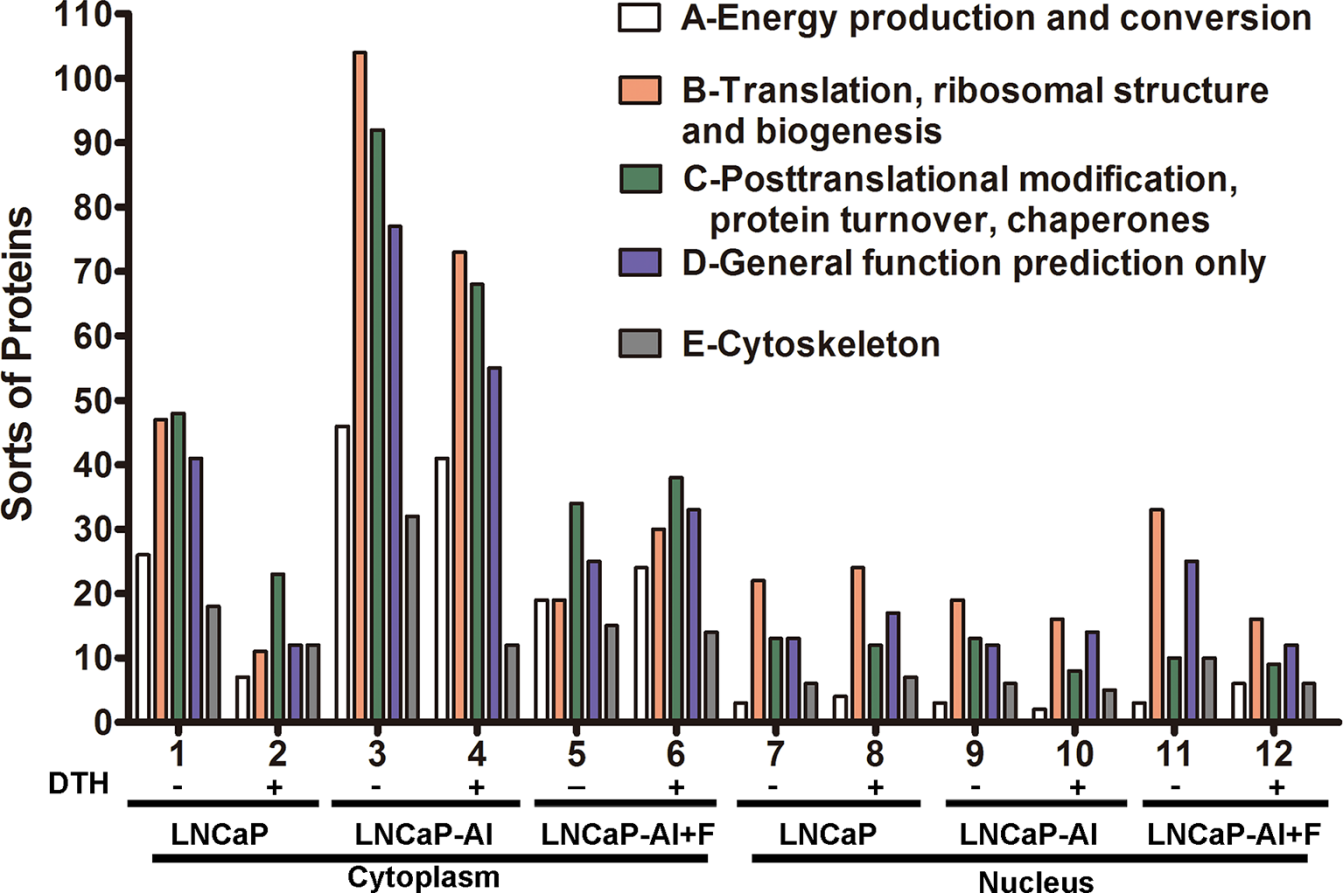
COG functional annotation of the AR pull-down proteins in the three PCa cells. The x-axis indicates different classification groups, and the y-axis indicates the number of proteins in each COG class. **(A–E)** represent the five most abundant COG categories: **(A)**-energy production and conversion; **(B)**-translation, ribosomal structure and biogenesis; **(C)**-posttranslational modification, protein turnover, and chaperones; **(D)**-general function prediction only; and **(E)**-cytoskeleton.

To deduce the functionality and biological processes associated with the identified AR pull-down proteins, Clusters of Orthologous Groups (COG) annotations were performed. The top 5 amount of proteins in COG classes were as follows: A-energy production and conversion; B-translation, ribosomal structure and biogenesis; C-posttranslational modification, protein turnover, and chaperones; D-general function prediction only; and E- cytoskeleton (**Figure 4**). As for cytoplasmic AR pull-down proteins, compared with LNCaP cells, the number of five COG functional proteins increased in LNCaP-AI cells while decreased in LNCaP-AI+F cells. It is observable from the results that an overall reduction number of cytoplasmic AR cofactors in LNCaP and LNCaP-AI cells when treated with DHT, but not in LNCaP-AI+F cells. It demonstrates that DHT promotes cytoplasmic AR dissociates with coregulators and imports to the nucleus in hormone-sensitive cells. Interestingly, in the cytoplasm of LNCaP-AI cells, 32 kinds of cytoskeleton-related proteins were enriched in AR-pulled down complex without DHT treatment, among which 15 only presented in this sample yet dissociated with AR after DHT treatment. The 15 proteins are as follows: ACTA1, ACTR1A, ACTR3, FLNA, FLNB, KIF5B, KIF15, LCP1, MAPRE1, MYH10, MYH14, MYO1C, MYO6, SEPT4 and TUBB4 (**Supplementary Table 5**). We found that AR was associated with filamin A only in the cytoplasm of LNCaP-AI cells, whereas replaced by Src tyrosine kinase when DHT was added. In line with these observations that AR directly interacts with Src in the cytoplasm of epithelial cells, yet it binds with filamin A in mesenchymal cells; however, the binding with filamin A will be replaced by Src when mesenchymal cells are treated with even low amount of androgen (20). The number of cytoskeleton proteins became similar in both cytoplasm of LNCaP and LNCaP-AI cells when stimulated with DHT suggesting LNCaP-AI cells may evolved into mesenchymal cells that prevalently communication with cytoskeleton proteins in the hormone deprivation condition while resumed to epithelial cells once acquired hormone. Cytoskeletal proteins might be involved in enhanced AR nuclear translocation abilities in LNCaP-AI cells during long-term hormone deprivation, according to the results shown in **Figure 2**.

**Figure 5:**
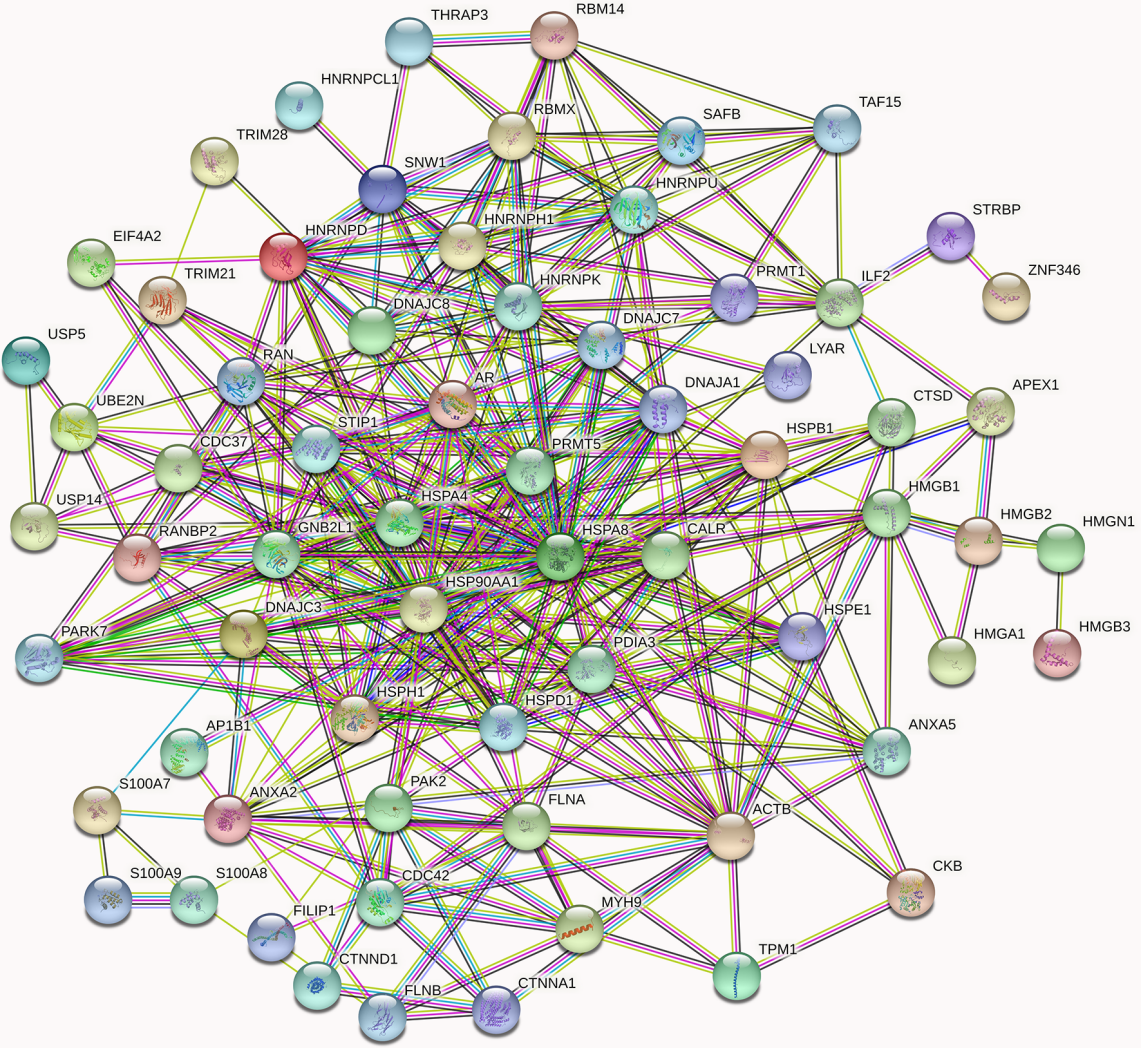
High-confidence protein-protein interaction network of already reported and AR new candidate coregulators. Overall, 67 proteins listed in **Supplementary Table 1** are involved in the AR signal networks. In the networks, links between proteins signify various interaction data supporting the network, colored by evidence type (see STRING website for color legend). These proteins that directly interact with AR have been verified by other articles, while the indirect interactions are putative AR cofactors. The nodes in different colors represent different proteins.

In addition, based on published studies together with protein-protein interaction analysis on the String (http://string.embl.de/) website, 73 different target proteins were identified in accordance with the protein score, molecular function, and metabolic pathways. These proteins were further categorized according to their function as heat shock proteins, cytoskeletal proteins, UBSs, RNA polymerase II-related proteins, transcription factors and other proteins that regulate the stability, translocation and transcription of AR. The proteins are listed in **Supplementary Table 1**, with the bold text indicating the known AR cofactors. A total of 27 confirmed AR coregulators were screened, among which 22 AR coactivators (**Supplementary Table 2**), such as HSP90, HSP70, THRAP3, NCOA1 and HMGB1, were identified (21–23). In addition, the 5 AR corepressors were HSP27, calreticulin, FLNA, SAFB and GNB2L2 (24–28). Moreover, a total of 46 proteins were screened as candidates for AR cofactors (**Supplementary Table 3**). In addition, dissecting the complex network of protein-protein interactions (PPIs) could facilitate a better understanding of the molecular mechanism of AR signaling pathways. Therefore, 67 proteins listed in **Supplementary Table 1** were also shown in **Figure 5** constructed by the String website in an evidence model. This enrichment indicates that the proteins that are tightly biologically connected are involved in AR signaling pathways as a group.

Since MYH9 obtains the highest protein score (1914.82) in MS identification among the 46 candidates of AR interactors, we suggest that MYH9 might be a novel binding protein of AR. On the other hand, as shown in **Figure 5**, MYH9 interacts with ACTB, FLNA and CDC42, which also interact with AR, suggesting that MYH9 may indirectly interact with AR. In fact, previous studies have provided evidence that several short sequence motifs in AR coregulators mediating specific interactions with AR, such as LxxLL (where L is leucine and x is any amino acid), are essential for mediating specific interactions with AR (6). Coincidentally, searching the MYH9 amino acid series, an LDDLL (where L is leucine and D is aspartic acid) motif was found. We therefore postulated that the LDDLL motif was responsible for mediating the AR–MYH9 interaction. Our speculation needs further rigorous experimental confirmation.

### The MYH9 Cytoskeletal Protein Is a Novel AR Cofactor

**Figure 6:**
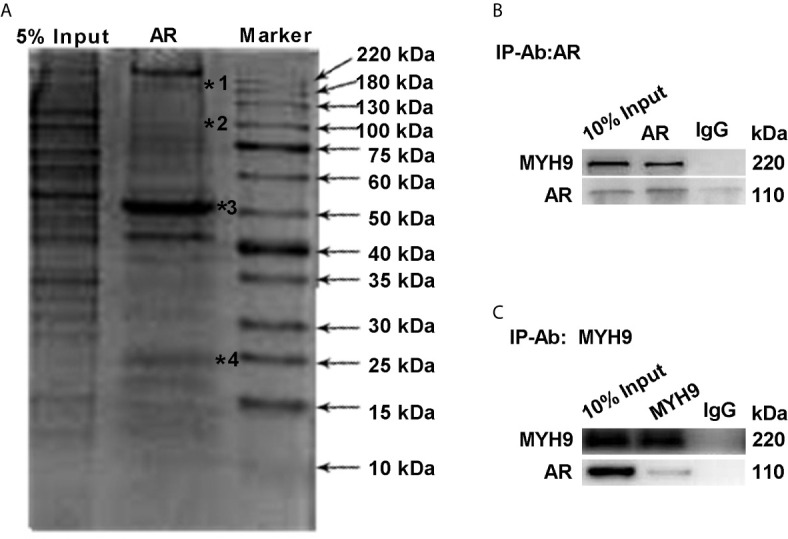
MYH9 interacts with the AR. **(A)** AR pull-down proteins were separated by electrophoresis and stained with Coomassie Brilliant Blue R250. The arrows indicate the molecular weight of the protein ladder, and the stars indicate proteins in the gel identified by MS. *1 to *4 represent MYH9, AR, IgG heavy chains and IgG light chains in sequence. **(B, C)** The endogenous interacting AR and MYH9 were coimmunoprecipitated and detected by WB with their respective antibodies using LNCaP-AI cells.

To further determine the interaction between AR and MYH9, lysates of LNCaP-AI cells were pulled down by CO-IP assay and stained with Coomassie Brilliant Blue. As **Figure 6A** displays, whole lysates of LNCaP-AI cells were coimmunoprecipitated, and then the eluate was electrophoresed on a 12% SDS-PAGE gel. The upper band shown in **Figure 6A** (marked by a star) was cut for MS identification. The proteins marked by a star in **Figure 6A** are MYH9, AR, IgG heavy chains and IgG light chains. To verify whether MYH9 identified by MS interacts with AR, we used an AR antibody for immunoprecipitation and MYH9 antibody for immunoblotting. In the lysate of LNCaP-AI cell, MYH9 was pulled down by AR but not by control IgG (**Figure 6B**). In addition, we carried out the experiment in a reciprocal manner using a MYH9 antibody for immunoprecipitation and an AR antibody for immunoblotting to confirm the physical interaction (**Figure 6C**). We observed an interaction between the two proteins in LNCaP-AI cells.

### The Nuclear Translocation of MYH9 Was Negatively Regulated by AR

**Figure 7:**
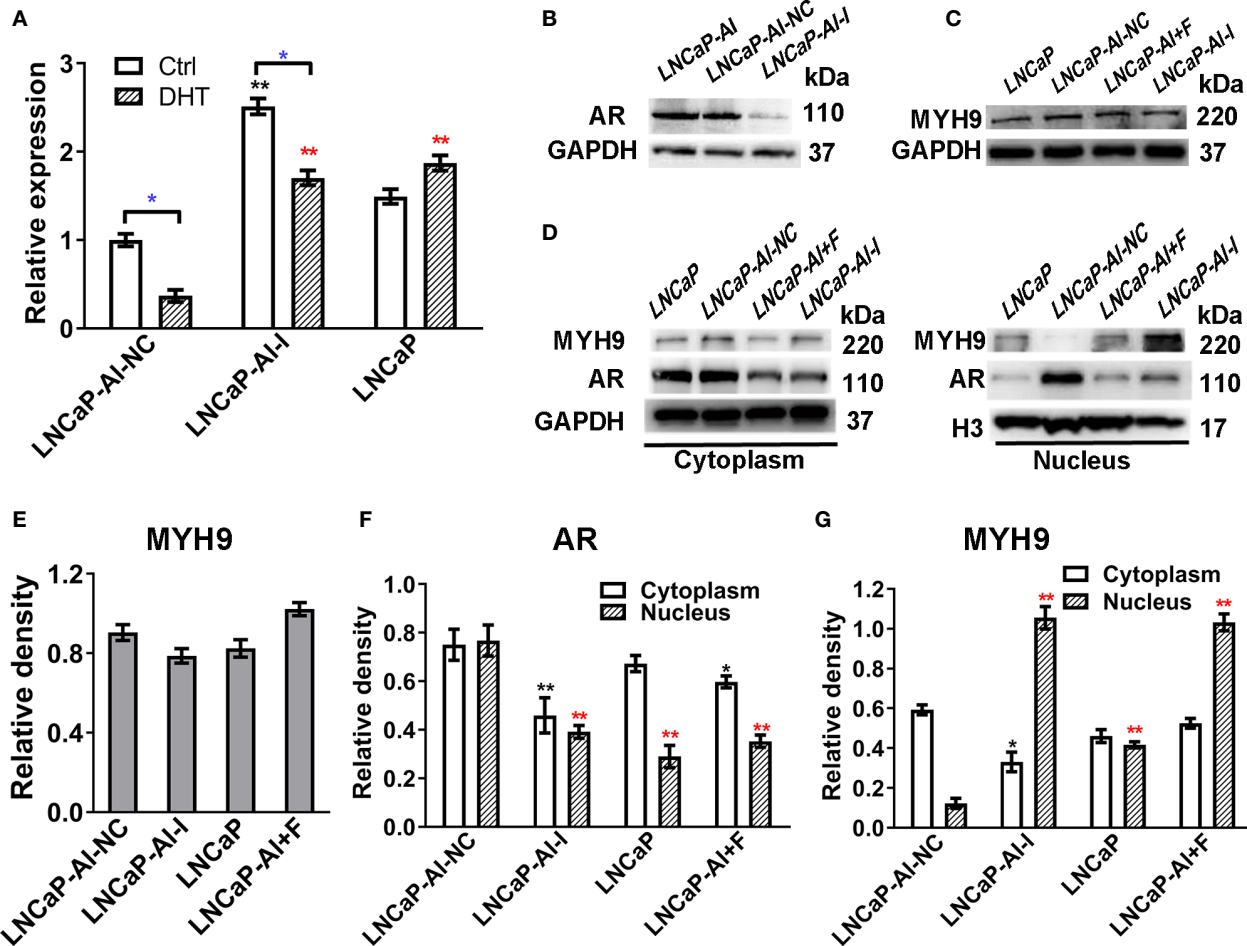
The nuclear translocation of MYH9 was enhanced after AR interference. **(A)** qRT-PCR analysis of MYH9 mRNA was assessed following serum starvation for 48 h before treatment with 10 nM DHT or equal amounts of ethanol for 24 h in the four cell lines. The results are expressed as the mean ± S.D. of three biological replicates, and * indicates a significant difference from the controls (*, P<0.05; one-way ANOVA followed by LSD multiple comparison test and T test for the same cell line treated with alcohol or 10 nM DHT). LNCaP-AI cells were transfected with AR shRNA (LNCaP-AI-I) or scrambled lentiviral particles (LNCaP-AI-NC), and lysates were blotted for AR **(B)** and MYH9 **(C)**. **(D)** MYH9 or AR nuclear and cytoplasmic fractions were blotted for detection. **(E)** The density of each MYH9 band in three independent experiments of **(C)** was determined. **(F, G)** The density of AR and MYH9 in three independent experiments of **(D)** was determined by ImageJ 1.52i software (National Institutes of Health, USA) and AR or MYH9 levels were normalized to GAPDH (cytoplasm) or H3 (nucleus) (*P < 0.05, **P < 0.01; one-way ANOVA followed by Dunnett’s test compared with LNCaP-AI-NC cells, n=3). The experiment was performed in triplicate and a representative experiment is shown.

We have verified that MYH9 is a novel AR cofactor; therefore, it is necessary to determine whether AR and MYH9 regulate each other, especially in AR nuclear translocation, and what role MYH9 plays in AR signaling pathways. We previously constructed AR-knockdown LNCaP-AI cells with AR shRNA lentivirus vectors (LNCaP-AI-I) or scrambled lentiviral particles (LNCaP-AI-NC). For the purpose of understanding MYH9 expression in ADPC and AIPC cells, both mRNA and protein levels were tested. MYH9 mRNA was systematically upregulated in LNCaP-AI-I cells compared with that in LNCaP-AI-NC cells, and MYH9 mRNA was decreased in both LNCaP-AI-NC cells and LNCaP-AI-I cells but not in LNCaP cells when stimulate with 10 nM DHT (**Figure 7A**). Unexpectedly, although the total MYH9 protein without DHT treatment was slightly increased in LNCaP-AI-I and LNCaP-AI+F cells, it was not significantly different among the four PCa cell lines (**Figures 7C, E**), which was similar to the total AR in LNCaP, LNCaP-AI cells (**Figures 1C, D**). We have performed a genome-wide analysis of androgen receptor binding sites in LNCaP and LNCaP-AI cells (29), and MYH9 was not discovered to be a AR target gene (date not shown). We observed that both nuclear and cytoplasmic AR was reduced when AR was knocked down (**Figures 7D, F**). More interestingly, MYH9 protein was more concentrated in the cytoplasm in LNCaP-AI-NC cells compared to LNCaP cells. Nonetheless, interference of AR disturbed MYH9 cellular distribution rather than changed its protein expression (**Figures 7D, G**). The present data suggest that interference with AR results in enhanced MYH9 nuclear translocation. Therefore, we speculate that MYH9 is a corepressor of AR.

### MYH9 Is a Corepressor of AR

**Figure 8:**
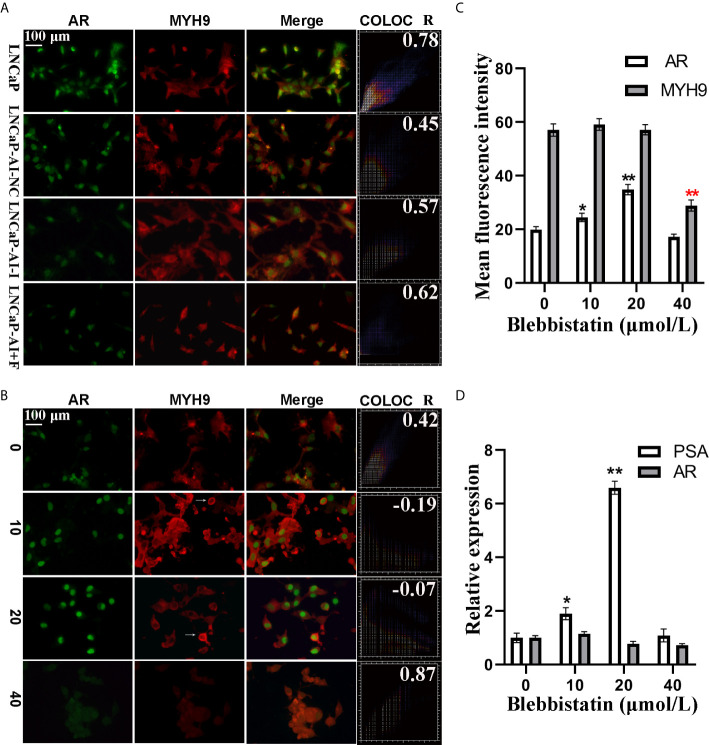
AR nuclear translocation was retarded by MYH9 in LNCaP-AI cells. **(A)** AR (green) and MYH9 (red) were colocalized in the four PCa cells, as detected by immunofluorescence. **(B–D)** LNCaP-AI cells were treated with blebbistatin at 0, 10, 20 and 40 μM for 2 h. The subcellular localization of AR (green) and MYH9 (red) was visualized using fluorescence microscopy **(B)**. The mean fluorescence intensity of AR and MYH9 in **(B)** was calculated and presented in **(C)**. AR and MYH9 mRNA expression treated with blebbistatin was detected by qRT-PCR and shown in **(D)**. The results are expressed as the mean ± SE of three biological replicates. The arrow pointing to MYH9 indicates its assembly in the perinuclear area. The scale bar in the upper left corner is 100 μm (40×). COLOC presents AR and MYH9 colocalization and R represents Pearson’s R value (above threshold); *P<0.05, **P<0.01; one-way ANOVA followed by Dunnett’s test compared with the blebbistatin untreated group, n=3.

Moreover, the function of MYH9 in AR translocation was investigated. To test whether MYH9 colocalized with AR, both the subcellular localization of AR and MYH9 was visualized using fluorescence microscopy followed by colocalization analysis in the four PCa cell lines (**Figure 8A**). We discovered that AR and MYH9 were more likely to colocalization in LNCaP cells with a highest Pearson’s R correlation coefficient (0.78). To test whether MYH9 inhibited AR nuclear localization, LNCaP-AI cells were treated with different concentrations of blebbistatin, a potent selective adenosine triphosphatase (ATPase) inhibitor of myosin II, and visualized using fluorescence microscopy (**Figure 8B**). As expected, treated with blebbistatin, Pearson’s R value of AR vs MYH9 colocalization in LNCaP- AI cells declined from 0.42 to -0.07. In addition, Pearson’s R value of AR vs DAPI colocalization increased from 0.80 to 0.99 (**Supplementary Figure 1A**). Besides, MYH9 was arrested in the cytoplasm by blebbistatin in a concentration-dependent manner. The concrete manifestation is a low concentration of blebbistatin (no more than 20 μmol/L) aggregating MYH9 in the cytoplasm while preventing MYH9 shuttling in the nucleus (**Supplementary Figure 1B**). Otherwise, a high concentration of blebbistatin (40 μmol/L) obviously changed the cell morphology (date not shown) and suppressed AR nuclear translocation (**Supplementary Figure 1A**, Pearson’s R value of AR vs DAPI colocalization was 0.23**)** and reduced MYH9 protein expression (**Figure 8C**). Conversely, AR nuclear translocation was promoted by blebbistatin (no more than 20 μmol/L). To further confirm the fluorescence assays, it was necessary to confirm whether MYH9 modulated the expression of AR target genes. PSA, a typical AR target gene, was analyzed by qRT-PCR. In accordance with the fluorescent assays, PSA mRNA was increased (6.6-fold) by blebbistatin treatment in a concentration-dependent manner but retarded at 40 μmol/L (**Figure 8D**). We observed that there was no effect of blebbistatin on AR mRNA expression (**Figure 8D**), further supporting the idea that MYH9, a corepressor of AR, attenuates AR nuclear translocation.

## Discussion

ADT has long been the primary treatment for advanced PCa. Unfortunately, once PCa cells survive from ADT leading to the emergence of CRPC. In recent years, second-generation androgens targeted inhibitors, abiraterone and enzalutamide, are first-line therapy options in CRPC, and they have been approved for upfront use with ADT in patients with metastatic androgen-dependent PCa. However, almost all CRPC patients finally progress to resistance as well (30). Exploring the mechanisms underlying CRPC is essential for settling this disease. We previously established the androgen-independent cell line LNCaP-AI through long-term androgen deprivation of LNCaP cells and confirmed that LNCaP-AI cells were more proliferative and aggressive than LNCaP cells (18). Mechanisms underlying AR reactivation in CRPC include AR gene overexpression/amplification, AR point mutations, AR splice variants and intratumoral androgen biosynthesis (31). However, these mechanisms are not sufficient to explain why DHT-independent cells are more malignant than DHT-dependent cells. Despite higher AR mRNA expression in AIPC cells (**Figure 1B**), the AR protein unexpected has no significant difference between LNCaP and LNCaP-AI cells (P = 0.07), even reduced in LNCaP-AI+F cells (P=0.01) (**Figures 1C, D**). Besides, the more intriguing phenomenon was a reduction of AR mRNA expression while an elevation of PSA expression in LNCaP-AI cells treated with DHT (**Figures 1A, B**). While as for LNCaP-AI+F cells, inverse results were obtained. It is common that discordant mRNA and protein expression (32, 33) and the discordant expression of AR and PSA (34), the mechanism of which needs further investigation. We further observed DHT promoting AR nuclear translocation in both LNCaP and LNCaP-AI cells, but failed in LNCaP-AI+F cells (**Figure 2**). Notwithstanding, AR translocation in LNCaP-AI+F cells were insensitivity to DHT, the nucleus/cytoplasm ratios were significantly higher than LNCaP cells treated with ethanol (**Figure 2E**). We speculate that AR is sequestered in the nuclear matrix by hydroxyflutamide therefore unable to bind to androgen-response elements (ARE) (35). To conclude, multiple lines of evidence demonstrated that not AR expression, but protein localization within the cell cytoplasm and nucleus was responsible for the differential expression of PSA.

Various determining factors are linked to AR nuclear translocation and are thus involved in PCa progression. The most common of which are AR-associated coregulators. For instance, ING3 interacts with AR and promotes AR acetylation and nuclear localization, which contributes to PCa cell growth and migration (36). Conversely, it is remarkable that the down-regulation of INPP4B changes neither AR protein nor mRNA expression, whereas INPP4B stimulates AR nuclear translocation as well as accelerates AR transcriptional activity, eventually leading to PCa cells survival from castration therapies (37). As a consequence, using CO-IP coupled with LC-MS/MS, we successfully identified and screened 73 different proteins as AR cofactors (**Supplementary Table 1**), among which MYH9 was identified as a novel cofactor of AR. The interaction between MYH9 and AR was confirmed using CO-IP and MS assays. In fact, several cytoskeletal proteins, such as filamin A (25), HMGB-1 and HMGB-2 (22), have been identified as AR cofactors and regulate AR activity. In line with our results, these three AR interactors were also identified as AR pull-down proteins (**Figure 5**). On the one hand, despite MYH9 mRNA was increased, the nucleus portion rather than total MYH9 protein was increased by AR interference in the LNCaP-AI cells (**Figure 7**). Rac1, stimulated by the AR/filamin A complex (38), will be reduced by AR silence. We speculate that repressed Rac1 activity results in reduction of cytoplasmic F-actin networks (39) which further dampens extranuclear MYH9 activity and enhances nuclear MYH9 function. All of these results indicate that the modulation of MYH9 expression by AR is spatiotemporal. More interestingly, MYH9, simultaneously acting as a carter, facilitates a variety of proteins shutting between the cytoplasm and nucleus, such as increases MICAL2 (molecules interacting with CasL 2) nuclear export and promotes tannexin 1 and CXCR4 (cysteine (C)-X-C receptor) nuclear import (40–42). The colocalization analysis suggest that blebbistatin-mediated depression in nuclear MHY9 result in an enhanced AR nuclear translocation capability (**Supplementary Figure 1**) and a decline in PSA mRNA expression in LNCaP-AI cells (**Figure 8D**). In addition, hypofunction of MYH9 leading to reduced AR nuclear translocation rather than AR expression. These observations suggesting that nuclear MYH9 facilitates AR export to the cytoplasm or cytoplasmic MYH9 restrains AR nuclear import. However, whether MYH9 interacts with AR directly or indirectly by other cytoskeletal proteins needs further investigation. The proposed mechanism of MYH9 in the modulation of AR trafficking will be determined in further experiments.

MYH9 belongs to the myosin superfamily, which is closely associated with proliferation, migration, invasion and metastasis of cancer (43). Many studies propose that MYH9 promotes the progression of many tumors, yet extensive investigations have obtained the strongest evidence that it serves as a tumor suppressor (43). Qing Liao et al. identified MYH9 as a direct target of LIM kinase 1 (LIMK1) and found that it is indispensable for LIMK1-mediated proliferation and migration in colorectal cancer (CRC) (44). In addition, it has been reported that the activated SRF/MYH9 axis induces gastric cancer (GC) invasion and metastasis, which is related to poor outcome (45). Moreover, MYH9 modulates EMT mediated by β-catenin to facilitate the proliferation, migration and invasion of pancreatic cancer (PC) cells (46). However, MYH9 haploinsufficiency induces invasive lobular carcinoma (ILC) formation (47). Interestingly, the loss of MYH9 in the heart and the tongue epithelium contribute to the progression of tongue invasive squamous cell carcinoma (ISCC) in a mouse model (48). Accordingly, MYH9 suppression of the head and neck progression of human squamous cell carcinomas (SCCs) through p53 activation was found to be compromised and reduced in SCCs with poor survival (49). In PCa cells, the status of MYH9 is also controversial, some studies indicated that MYH9 was significantly upregulated in PCa compared to benign prostate hyperplasia samples through quantitative proteomics (50). Conversely, MYH9 was found to be downregulated in the extracapsule of aggressive prostate cancers versus organ-confined disease phenotypes (51). In the present study, although the expression of MYH9 was not significantly different between LNCaP and LNCaP-AI cells, it increased in the cytoplasm while decreased in the nucleus of LNCaP-AI cells. Nuclear MYH9 acts as a transcription factor and binds to the promoter of CTNNB1 (52), suggesting the function of nuclear MYH9 is different from the common cytoplasmic ones that acts as scaffold protein promoting cell migration and invasiveness. We speculate that increased cytoplasmic MYH9 interacts with F-actin and other cytoskeleton proteins promoting cell migration and invasiveness while decreased levels of nuclear MYH9 reduce nuclear p53 accumulation. Besides, the nuclear retention of AR leads to enhanced cell growth. Taken together, the abnormal distribution of MYH9 and AR may contribute to the transformation of hormone-sensitive LNCaP cells to hormone-insensitive LNCaP-AI cells. Nevertheless, the function of MYH9 in the progression of PCa and AIPC remains elusive and warrants further investigation.

In conclusion, we demonstrate that MYH9 functions as a novel AR corepressor. This notion is supported by the finding that MYH9 retards the transcriptional activity of AR in PCa cells. Moreover, we suggest that MYH9 is a key cytoskeletal protein involved in AIPC transformation, indicating that MYH9 is a potential therapeutic target in PCa.

## Data Availability Statement

The original contributions presented in the study are included in the article/**Supplementary Material**. Further inquiries can be directed to the corresponding authors.

## Author Contributions

WL and ZT designed the study. CL, ZL, XD, PY, and KP performed the experiments. WL and CL wrote the manuscript. All authors contributed to the article and approved the submitted version.

## Funding

This study was supported by grant from the National Natural Science Foundation of China Youth Science Foundation Project (Grant nos. 81802571), and Zhejiang Medical and Health Science and Technology Project (2019RC039).

## Conflict of Interest

The authors declare that the research was conducted in the absence of any commercial or financial relationships that could be construed as a potential conflict of interest.

## Abbreviations

ADPC, androgen-dependent prostate cancer; ADT, androgen deprivation therapy; AIPC, androgen-independent prostate cancer; AP, affinity chromatography; AR, androgen receptor; ARA, androgen receptor-associated protein; ARE, androgen-response elements; COG, cluster of orthologous groups; CO-IP, coimmunoprecipitation; CRC, colorectal cancer; CRPC, castrate resistant prostate cancer; CS−FBS, charcoal stripped fetal bovine serum; CXCR4, cysteine (C)-X-C receptor; CYP, cytochrome P450; DAPI, 4,6-diamidino-2-phenyl-indole; DHT, dihydrotestosterone; DIGE, differential in-gel electrophoresis; F, hydroxyflutamide; FBS, fetal bovine serum; FLNA, filamin A; GC, gastric cancer; GNB2L, guanine nucleotide-binding protein subunit beta-2-like; HSP, heat-shock protein; HMGB, high mobility group box B; ILC, invasive lobular carcinoma; LC-MS, liquid chromatography coupled with tandem mass spectrometry; IGF1, insulin-like growth factor-1; ISCC, invasive squamous cell carcinoma; LIM-1, LIM kinase 1; MICAL2, molecules interacting with CasL 2; MYH9, myosin heavy chain 9; NCOA, nuclear receptor coactivator; NCoR, nuclear receptor corepressor; PC, pancreatic cancer; PCa, prostate cancer; PLCε, phospholipase C epsilon; PSA, prostate specific antigen; SAFB, scaffold attachment factor B; SGK1, serum and glucocorticoid-induced protein kinase 1; shRNA, short hairpin ribonucleic acid; SgI, semenogelin I; SLIRP, SRA stem-loop interacting RNA binding protein; SMRT, thyroid hormone receptor; SRC, steroid receptor coactivator; THRAP, thyroid hormone receptor-associated protein.
