# Supplementary material for: The MYH9 Cytoskeletal Protein Is a Novel Corepressor of Androgen Receptors
Source: Front Oncol. 2021 Apr 1;11:641496. doi: 10.3389/fonc.2021.641496 (PMC8093144; doi:10.3389/fonc.2021.641496)
Supplement: Supplementary file 1 [file DataSheet_1.docx]

| 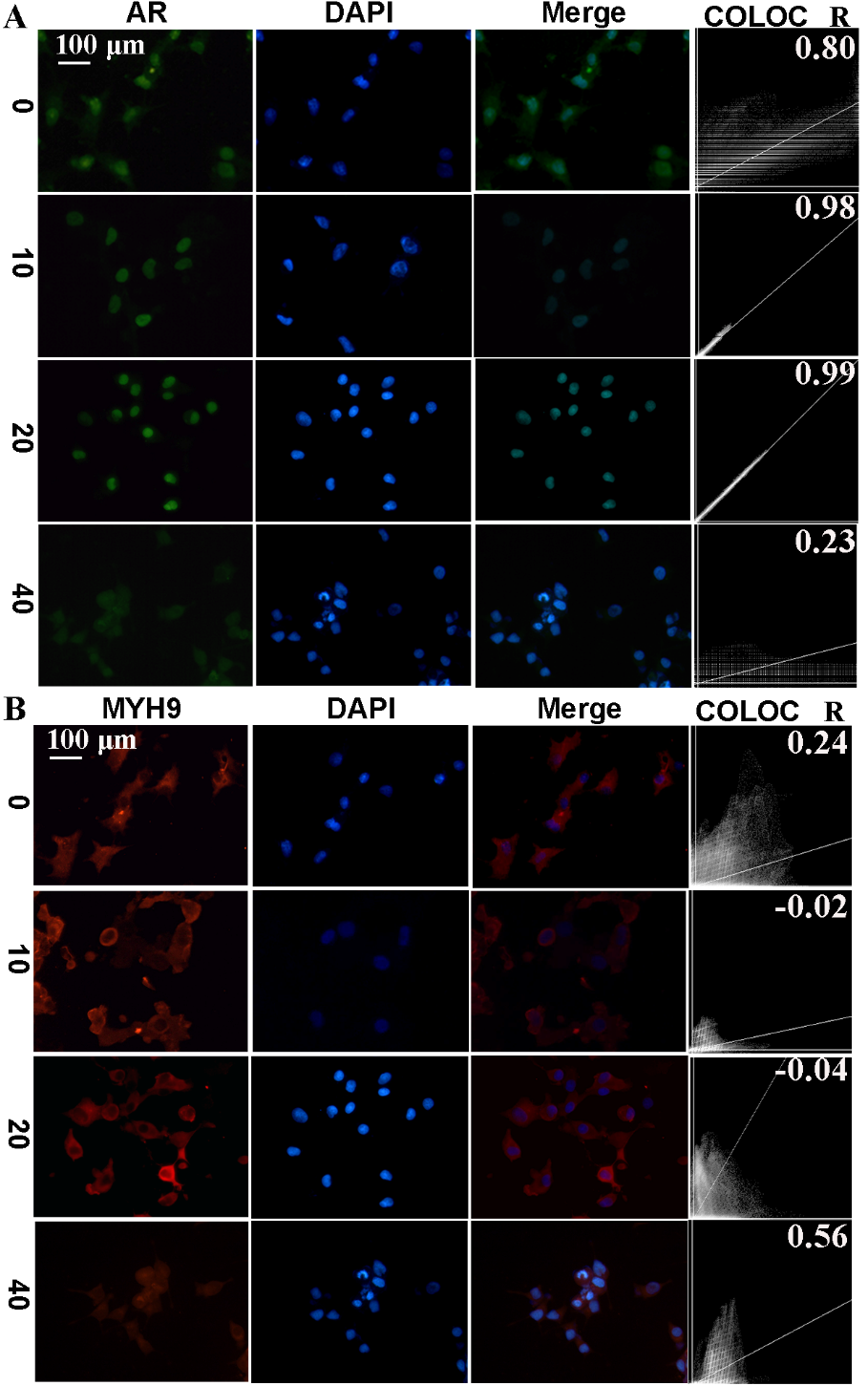 |
| --- |
| Supplementary Fig. 1 AR nuclear translocation was retarded by MYH9 in LNCaP-AI cells. LNCaP-AI cells were treated with blebbistatin at 0, 10, 20 and 40 μM for 2 h. The subcellular localization of AR (green), MYH9 (red) and DAPI (blue) was visualized using fluorescence microscopy. The scale bar in the upper left corner is 100 μm (40×). COLOC presents AR vs DAPI (A) or MYH9 vs DAPI(B) colocalization. R represents Pearson's R value (above threshold). |
