# Supplementary material for: The MYH9 Cytoskeletal Protein Is a Novel Corepressor of Androgen Receptors
Source: Front Oncol. 2021 Apr 1;11:641496. doi: 10.3389/fonc.2021.641496 (PMC8093144; doi:10.3389/fonc.2021.641496)
Supplement: Supplementary file 2 [file Table_1.docx]

| **Table-1 AR cofactors in AR pull-down proteins** | |
| --- | --- |
| Name of proteins | comments |
| HSP |  |
| HSPD1 60 kDa heat shock protein mitochondrial | Interacts with HSP90AA, HSPA4, HSPE1 |
| HSPA8 Isoform 1 of Heat shock cognate 71 kDa protein | Interacts with HSP40, HSP90AA |
| **HSPA5 78 kDa glucose-regulated protein** | alternate name HSP70 protein |
| **HSP90AA1 Isoform 2 of Heat shock protein HSP 90-alpha** | AR coactivator |
| **HSPA9 Stress-70 protein mitochondrial** | AR coactivator |
| **HSP90B1 Endoplasmin** | AR coactivator |
| **HSPA1B; HSPA1A Heat shock 70 kDa protein 1A/1B** | AR coactivator |
| **HSP90AB1 Heat shock protein HSP 90-beta** | AR coactivator |
| **HSPB1 Heat shock protein beta-1** | Alternate name HSP27, AR corepressor |
| HSPE1 10 kDa heat shock protein mitochondrial | Interacts with HSPD1 |
| **HSPA4 Heat shock 70 kDa protein 4** | heat shock 70kDa protein 4，Interacts with HSP90AA |
| HSPH1 Isoform Beta of Heat shock protein 105 kDa | Interacts with HSP90AA1, HSPA8, UBC |
| **CDC37 HSP90 co-chaperone Cdc37** | Interacts with HSP90AA |
| Cytoskeleton |  |
| **HMGB1 High mobility group protein B1** | AR coactivator |
| **HMGB2 High mobility group protein B2** | AR coactivator |
| HMGB3 High mobility group protein B3 | Combines with single chain of DNA, similar function with HMGB1/HMGB2，also combines with HMGA1 |
| **HMGA1 Isoform HMG-Y of High mobility group protein HMG-I/HMG-Y** | Combines with CREBBP, AR coactivators |
| HMGN1P38; HMGN1 Non-histone chromosomal protein HMG-14 | Interacts with HSP70, ER1and EP300 |
| FILIP1 Isoform 1 of Filamin-A-interacting protein 1 | Combines with FILA |
| FLNB Isoform 1 of Filamin-B | Interacts with FLNA |
| **FLNA Isoform 2 of Filamin-A** | AR corepressor |
| CTNNA1 Isoform 1 of Catenin alpha-1 | Interacts with β-catenin |
| CTNND1 Isoform 1AB of Catenin delta-1 | Interacts with β-catenin |
| **ACTB Actin cytoplasmic 1** | Join in AR translocation indirectly |
| TPM1 tropomyosin alpha-1 chain isoform 2 | Interacts with actin-filaments |
| MYH9 Isoform 1 of Myosin-9 | Cytoskeleton, Interacts with Mst1(S100A4), CDK4, CDK6, TP53 |
| Ubiquitin | UBC, AR coactivator |
| **TRIM21 Isoform 1 of E3 ubiquitin-protein ligase TRIM21** | Interacts with UBC |
| RPS27A Ubiquitin-40S ribosomal protein S27a | Interacts with UBC |
| **UBE2N Ubiquitin-conjugating enzyme E2 N** | Interacts with UBC |
| USP14 Ubiquitin carboxyl-terminal hydrolase 14 | Interacts with PSMD3, PSMD4, PSMD6, PSMD12, PSMD14（all have been identified in AR pull-down proeteins） |
| USP5 Isoform Long of Ubiquitin carboxyl-terminal hydrolase 5 | Interacts with UBC, UPS26 is AR coactivator |
| **DNAJ** | HSP40 (dnaJ, ydj1p), AR coactivator |
| DNAJC8 DnaJ homolog subfamily C member 8 | Homology of HSP40, combines to RNA polymerase II |
| DNAJC7 dnaJ homolog subfamily C member 7 | Homology of HSP40, Interacts with HSP90AA1, UBC |
| DNAJA1 DnaJ homolog subfamily A member 1 | Interacts with HSP70, HSP90AA, UBE2D3 |
| DNAJC3 DnaJ homolog subfamily C member 3 | Interacts with EIF2AK2, EIF2AK3, EIF2AK4, HSP70 |
| Translation function proteins |  |
| SNORA63 Isoform 1 of Eukaryotic initiation factor 4A-II | Fuction as AR transcription with RNA polymerase II |
| EEF1A1 Elongation factor 1-alpha 1 | Fuction as AR transcription with RNA polymerase II |
| EEF2 Elongation factor 2 | Fuction as AR transcription with RNA polymerase II |
| **SUB1 Activated RNA polymerase II transcriptional coactivator p15** | RNA polymerase II coactivator |
| Heterogeneous nuclear ribonucleoprotein |  |
| **HNRNPK Isoform 1 of Heterogeneous nuclear ribonucleoprotein K** | AR coactivator |
| HNRNPH1 Heterogeneous nuclear ribonucleoprotein H | Interacts with UBC |
| HNRNPCL1 Heterogeneous nuclear ribonucleoprotein C-like 1 | Interacts with EIF2AK2 |
| HNRNPU Isoform Short of Heterogeneous nuclear ribonucleoprotei | [Interacts with HNRNPK, RNA polymerase II](http://www.string-db.org/newstring_cgi/display_single_node.pl?taskId=iLpP1B6Pk8iz&node=991370&targetmode=proteins) |
| RBMX Heterogeneous nuclear ribonucleoprotein G | Interacts with UBC, HNRNPK |
| HNRNPD Isoform 1 of Heterogeneous nuclear ribonucleoprotein D0 | Interacts with EIF4G1, HNRNPK |
| **CALR Calreticulin** | AR corepressor |
| **SNW1 Nuclear receptor coactivator NC0A-62** | NCOA1, NCOA2, NCOA3, NCOA4 were AR coactivator |
| **AP1B1 Isoform A of AP-1 complex subunit beta-1** | AP-1, AR coactivator |
| **SAFB Scaffold attachment factor B1** | AR corepressor |
| **PARK7 Protein DJ-1** | DJ-1, AR coactivator |
| **CDC42** **Isoform 2 of Cell division control protein 42 homolog** | Alternate name P21，Interacts with PAK6, PAK7 |
| ANXA2 Isoform 2 of Annexin A2 | Combines with CDC42 |
| ANXA5 Annexin A5 | Combines with vimentin |
| PAK 2 Serine/threonine-protein kinase PAK 2 | PAK6 and PAK7，AR coactivator, Interacts with CDC42 |
| **TAF15** | AR coactivator |
| **PRMT1 protein arginine N-methyltransferase 1 isoform 3** | Interacts with TAF15, POLR2E, SAFB, TBP, CBP |
| C1orf116 Isoform 2 of Specifically androgen-regulated gene protein | Interacts with Smad3, Smad2（AR coactivators） |
| **SELENBP1 Isoform 1 of Selenium-binding protein 1** | AR coactivator，Interacts with TAF15 |
| CTSD Cathepsin D | Down-regulated expression in AIPC, Interacts with ER, TP53 |
| PDIA3 14 kDa protein | Interacts with HSP70 |
| CKB Creatine kinase B-type | Down-regulated expression in AIPC, Interacts with UBC |
| TIPRL Isoform 2 of TIP41-like protein | Tip60, AR corepressor |
| CDK1 Putative uncharacterized protein DKFZp686L20222 | CDK9, AR coactivator |
| APEX1 DNA-apurinic or apyrimidinic site) lyase | Interacts with EP300 and TP53 |
| TRIM28 Isoform 1 of Transcription intermediary factor 1-beta | TRIM24, TRIM68, AR coactivators |
| STRBP Isoform 2 of Spermatid perinuclear RNA-binding protein | Interacts with ZNF346, HNRNU, RNABP5, ILF2 |
| ILF2 Interleukin enhancer-binding factor 2 | Interacts with ZNF346, HNRNU, RNABP5, TRIM28 |
| RNA-binding protein | Interacts with ZNF346, HNRNU, STRBP, ILF2 |
| ZNF346 Isoform 2 of Zinc finger protein 346 | Interacts with TP53, EIF2AK2 |
| ZNF148 Zinc finger protein 148 | Interacts with TP53, Vimentin, HDAC1, HDAC3 |
| ZNF618 Isoform 2 of Zinc finger protein 618 | Function as transcription regulator |
| FUS Isoform Short of RNA-binding protein FUS | Interacts with HNRNPK, RNA polymerase II |
| **RBM14; RBM14-RBM4 Isoform 1 of RNA-binding protein 14** | Coactivator of nuclear receptor，recruits NCOA and CITED1, Interacts with CDK8 |
| **RANP1; RAN GTP-binding nuclear protein Ran** | Ran, AR coactivator |
| RANBP2 E3 SUMO-protein ligase RanBP2 | Interacts with RAN, RAB19, SUMO1, SUMO2（AR coactivators） |
| LYAR Cell growth-regulating nucleolar protein | Interacts with GNL2(guanine nucleotide binding protein-like 2 |
| **GNB2L1 Guanine nucleotide-binding protein subunit beta-2-like 1** | Coding RACK1, AR corepressor |
| **THRAP3 Thyroid hormone receptor-associated protein 3** | AR coactivator |
| TGM3 Protein-glutamine gamma-glutamyltransferase E | Interacts with S100A9 |
| S100A9 Protein S100-A9 | Interacts with TLR4, MAPK1, PARP1, S100A8 |
| S100A8 Protein S100-A8 | Interacts with TLR4, S100A9 |
| S100A7 Protein S100-A7 | Interacts with VEGFA |
| STIP1 Stress-induced-phosphoprotein 1 | Interacts with HSP90AA |
| PRMT5 protein arginine N-methyltransferase 5 | Interacts with TP53, H3, H4 |
