# Supplementary material for: The MYH9 Cytoskeletal Protein Is a Novel Corepressor of Androgen Receptors
Source: Front Oncol. 2021 Apr 1;11:641496. doi: 10.3389/fonc.2021.641496 (PMC8093144; doi:10.3389/fonc.2021.641496)
Supplement: Supplementary file 3 [file Table_2.docx]

| **Table-2 AR cofactors in AR pull-down proteins** | | | | | | | |
| --- | --- | --- | --- | --- | --- | --- | --- |
| Protein Name | Protein Score | Protein Mass | Coverage | Protein Name | Protein Score | Protein Mass | Coverage |
| **coactivators** | | | | **coactivators** | | | |
| HSPD1 | 2966.57 | 61187.45 | 56.54 | UBE2N | 77.3 | 17184 | 7.24 |
| HSP90 | 2418.98 | 98669.69 | 38.64 | AP1B1 | 71.33 | 105482.41 | 3.48 |
| HSP70 | 1943.93 | 72402.48 | 42.05 | CDC42 | 68.65 | 21587.15 | 14.14 |
| ACTB | 1339.08 | 42051.86 | 51.73 | THRAP3 | 65.95 | 108658.04 | 1.15 |
| HNRNPK | 573.9 | 51229.51 | 23.11 | TAF15 | 58.42 | 61749.08 | 1.53 |
| HMGB1 | 323.72 | 25049.23 | 24.19 | TRIM21 | 54.12 | 55161.51 | 5.89 |
| SUB1 | 145.95 | 14386.41 | 22.05 | CDC37 | 51.4 | 44953.19 | 2.91 |
| SELENBP1 | 141.37 | 52927.84 | 9.96 | PARK7 | 48.22 | 20049.55 | 11.11 |
| HMGB2 | 130.09 | 24189.79 | 17.7 | **corepressors** | | | |
| RANP1 | 113.4 | 24578.68 | 16.2 | GNB2L1 | 233.15 | 35510.73 | 23.03 |
| SNW1 | 97.62 | 43362.13 | 6.68 | Calreticulin | 222.94 | 48282.89 | 12.23 |
| RBM14 | 81.87 | 69619.97 | 2.84 | HSP27 | 198.08 | 22825.51 | 22.44 |
| PRMT1 | 81.82 | 26380.28 | 5.24 | SAFB | 115.54 | 103035.69 | 2.84 |
| HMGA1 | 80.32 | 10672.62 | 9.38 | FLNA | 114.46 | 282580.51 | 0.72 |
