# Supplementary material for: The MYH9 Cytoskeletal Protein Is a Novel Corepressor of Androgen Receptors
Source: Front Oncol. 2021 Apr 1;11:641496. doi: 10.3389/fonc.2021.641496 (PMC8093144; doi:10.3389/fonc.2021.641496)
Supplement: Supplementary file 4 [file Table_3.docx]

| **Table-3 Presumptive AR cofactors in AR pull-down proteins** | | | | | | | |
| --- | --- | --- | --- | --- | --- | --- | --- |
| Protein Name | Protein Score | Protein Mass | Coverage | Protein Name | Protein Score | Protein Mass | Coverage |
| MYH9 | 1914.82 | 227646.06 | 26.48 | ILF2 | 92.56 | 43263.27 | 9.74 |
| HSPA8 | 1885.48 | 71082.31 | 39.16 | RBMX | 83.92 | 42306.33 | 6.65 |
| PDIA3 | 1007.99 | 57145.9 | 36.24 | C1orf116 | 83.48 | 37614.82 | 16.34 |
| CKB | 936.1 | 42902.43 | 56.96 | TPM1 | 83.34 | 28491.57 | 8.98 |
| FLNB | 906.69 | 280156.87 | 13.18 | PRMT5 | 80.91 | 71902.19 | 1.61 |
| APEX1 | 532.04 | 35931.32 | 31.76 | USP14 | 69.87 | 56489.2 | 2.02 |
| LYAR | 440.86 | 44044.03 | 30.08 | HMGB3 | 64.82 | 23136.51 | 12.5 |
| HNRNPU | 348.98 | 89665.43 | 10.55 | HMGN1P38 | 63.98 | 10652.59 | 11 |
| ZNF346 | 261.02 | 36432.23 | 15.36 | HSPE1 | 63.82 | 10924.87 | 19.61 |
| SNORA63 | 257.05 | 46600.86 | 19.41 | TGM3 | 60.06 | 76925.71 | 1.59 |
| TRIM28 | 253.49 | 90261.08 | 11.86 | ANXA5 | 58.35 | 35971.42 | 7.5 |
| HSPH1 | 234.87 | 92969.79 | 9.09 | DNAJC7 | 57.28 | 50806.06 | 2.51 |
| CTNND1 | 222.78 | 107853.24 | 9.77 | RANBP2 | 56.22 | 362364.7 | 0.37 |
| CTNNA1 | 203.09 | 100692.78 | 14.02 | DNAJA1 | 55.61 | 45580.73 | 3.27 |
| CTSD | 184.93 | 45036.82 | 16.75 | DNAJC8 | 52.14 | 29823.42 | 7.91 |
| HNRNPH1 | 147.62 | 49483.52 | 10.24 | TIPRL | 51.08 | 20372.01 | 6.74 |
| STIP1 | 133.43 | 63226.64 | 12.34 | HNRNPCL1 | 47.44 | 32179.71 | 3.41 |
| ANXA2 | 131.91 | 40670.82 | 8.4 | ZNF618 | 40.69 | 97208.26 | 1.05 |
| S100A8 | 122.51 | 10884.67 | 31.18 | PAK 2 | 40.33 | 58324.05 | 1.71 |
| S100A7 | 118.3 | 11577.61 | 21.78 | USP5 | 37.12 | 96637.68 | 1.17 |
| HNRNPD | 116.76 | 38581.37 | 10.14 | DNAJC3 | 37.1 | 58000.34 | 1.79 |
| ZNF148 | 106.85 | 89547.82 | 2.14 | STRBP | 36.03 | 72605.3 | 1.98 |
| S100A9 | 97.39 | 13290.53 | 13.16 | FILIP1 | 33.4 | 138537.05 | 0.58 |
