# Supplementary material for: The MYH9 Cytoskeletal Protein Is a Novel Corepressor of Androgen Receptors
Source: Front Oncol. 2021 Apr 1;11:641496. doi: 10.3389/fonc.2021.641496 (PMC8093144; doi:10.3389/fonc.2021.641496)
Supplement: Supplementary file 5 [file Table_4.docx]

| **Table-4 Unique skeleton proteins in cytoplasmic AR pull-down proteins of LNCaP-AI cells** | |
| --- | --- |
| Name of proteins | comments |
| ACTA1 | Actin alpha skeletal muscle |
| ACTR1A | Alpha-centractin |
| ACTR3 | Actin-related protein 3 |
| FLNA | Isoform 2 of Filamin-A |
| FLNB | Isoform 1 of Filamin-B |
| KIF5B | Kinesin-1 heavy chain |
| KIF15 | Isoform 1 of Kinesin-like protein KIF15 |
| LCP1 | Plastin-2 |
| MAPRE1 | Microtubule-associated protein RP/EB family member 1 |
| MYH10 | Isoform 1 of Myosin-10 |
| MYH14 | Isoform 1 of Myosin-14 |
| MYO1C | Isoform 2 of Myosin-Ic |
| MYO6 | Myosin of class VI |
| SEPT4 | Isoform 4 of Septin-4 |
| TUBB4 | Tubulin beta-4 chain |
